# Disgusting odors trigger the oral immune system

**DOI:** 10.1093/emph/eoac042

**Published:** 2022-12-15

**Authors:** Stephanie Anja Juran, Arnaud Tognetti, Johan N Lundström, Lalit Kumar, Richard J Stevenson, Mats Lekander, Mats J Olsson

**Affiliations:** Department of Clinical Neuroscience, Karolinska Institutet, Stockholm, Sweden; Department of Clinical Neuroscience, Karolinska Institutet, Stockholm, Sweden; Department of Clinical Neuroscience, Karolinska Institutet, Stockholm, Sweden; Department of Women’s and Children’s Health, Karolinska Institutet, Stockholm, Sweden; Department of Psychology, Macquarie University, North Ryde, NSW, Australia; Department of Clinical Neuroscience, Karolinska Institutet, Stockholm, Sweden; Department of Psychology, Stockholm University, Stockholm, Sweden; Osher Center for Integrative Health, Department of Clinical Neuroscience, Karolinska Institutet, Stockholm, Sweden; Department of Clinical Neuroscience, Karolinska Institutet, Stockholm, Sweden

## Abstract

Recent research has characterized the behavioral defense against disease. In particular the detection of sickness cues, the adaptive reactions (e.g. avoidance) to these cues and the mediating role of disgust have been the focus. A presumably important but less investigated part of a behavioral defense is the immune system response of the observer of sickness cues. Odors are intimately connected to disease and disgust, and research has shown how olfaction conveys sickness cues in both animals and humans. This study aims to test whether odorous sickness cues (i.e. disgusting odors) can trigger a preparatory immune response in humans. We show that subjective and objective disgust measures, as well as TNFα levels in saliva increased immediately after exposure to disgusting odors in a sample of 36 individuals. Altogether, these results suggest a collaboration between behavioral mechanisms of pathogen avoidance in olfaction, mediated by the emotion of disgust, and mechanisms of pathogen elimination facilitated by inflammatory mediators.

Disgusting stimuli are associated with an increased risk of infection. We here test whether disgusting odors, can trigger an immune response in the oral cavity. The results indicate an increase level of TNFα in the saliva. This supports that disease cues can trigger a preparatory response in the oral cavity.

## INTRODUCTION

Upon detection of disgusting objects, a repertoire of autonomic and involuntary behavioral responses is initiated to avoid pathogen-rich objects like feces and rotten food, and to prepare for the possibility of contact. Pathogen avoidance offers a considerable evolutionary advantage by reducing the probability for disease contagion when compared with the exhaustive and cost-intensive mechanisms of pathogen elimination in form of a fully developed immune response [1, 2].

Pioneering work in this field has shown that mere viewing of disease relevant pictures, e.g. a sneezing person, when compared with threatening photographs, led to increased production of IL-6 (interleukin 6) in white blood cells when stimulated *in vitro* with lipopolysaccharide [3]. Follow-up studies extended these findings to salivary-bound immune reactions and viewing of disgusting pictures in general as opposed to pictures showing disease stimuli only, indicating disgust-related increase in salivary TNFα but decrease in sIgA [4, 5]. Having confirmed such interaction between pathogen avoidance (disgust) and elimination mechanisms (via IS) for the visual modality, the question if it is universal also for other senses remains to be addressed.

In the current study, we focus on the olfactory sense for two main reasons. We have already shown [6, 7] that human body odor contains an early perceptual cue of systemic inflammation that indicates that the human sense of smell is part of a behavioral defense against disease, often referred to as the behavioral immune system [8]. Secondly, olfaction is strongly linked to the emotion of disgust, and possibly to the mechanisms related to the oral immune response. We describe the relationship between olfaction and disgust in detail below.

### The relationship between olfaction and disgust

A special relationship between the emotion disgust and the chemical senses can be inferred from the idea that disgust evolved from distaste, i.e. the automatic response to expel unsavory and potentially harmful food from the mouth [1, 2] and accompanying facial expressions [9] such as gaping mouth, protruding tongue, narrowing nostrils and frowning of the eyebrows [10]. Based on these observations, the theory has emerged that the distaste-reflex generalizes to other disease related objects, perhaps via some form of behavioral conditioning or social learning [11]. Such transfer-learning may be especially relevant for odors due to the close connectivity between the olfactory sense and taste [12].

Two features of the facial disgust/distaste expression are so characteristic of the disgust emotion that they are widely used as objective indicators of disgust. Muscle activation from the regions around the nose (levator labii) and eyebrows (corrugator supercilii) can be reliably measured with electromyography (EMG) and have been shown to indicate occurrence of disgust after visual [13, 14] and olfactory stimulation [15, 16]. In the current study, we use these objective measures in parallel to subjective ratings to confirm olfactory disgust induction.

### Olfaction and oral immunity

Expression of salivary immune modulators occurs not only from salivary glands but also from taste buds [17]. Mouse taste buds responsive to sweet and umami taste, express TNFα that is upregulated in presence of bacteria and regulated by an antagonizing mechanism involving the expression of anti-inflammatory interleukin 10 (IL10) from other types of taste-buds expressing bitter-taste receptors [18]. Furthermore, the absence of TNFα has been shown to downregulate bitter taste perception in mice [19].

These findings indicate a relationship between oral immunity, our ability to perceive bitter taste, and thus potentially toxic nutrients, and systems regulating food intake. Furthermore, TNFα is part of the mechanisms that cause a ‘sickness response’, the coordinated behavioral response that involves, e.g. malaise, fatigue, pain, coldness and reduced appetite to save energy and help fever production in order to combat infection [20], and it has been ascribed a special role in anorectic food avoidance often occurring as part of the sickness response [21]. Thus, TNFα presents as a highly relevant oral immune modulator in relation to olfactory induced disgust.

Earlier studies showing upregulation of salivary TNFα in response to visual disgust, also showed downregulation of sIgA. This was interpreted as a conserving mechanism with the aim to avoid waste of sIgA in situations where disgust or distaste would initiate increased saliva secretion to flush the mouth from objectionable objects [4, 5].

With this background, the current study tests the hypotheses that odor-induced disgust will initiate the oral immune system as a part of a behavioral defense against disease. More specifically, we expect olfactory disgust to trigger the salivary reflex and thus the composition of salivary immune modulators by upregulation of the secretory cytokine TNFα and downregulation of the first line antibody sIgA.

## METHODS

This study was performed in the accordance with the declaration of Helsinki. All participants gave their informed consent in writing. The study was ethically approved by the Swedish authority for ethical approval (Etikprövningsmyndigheten: 014/229-31/4).

### Participants

Based on a previous published study that found a moderate effect size of disease-related stimuli exposure on the surge of salivary TNFα [5], a power analysis based on a paired *T*-test with 80% power, α = 0.05, and a moderate effect size *t* = 0.5, indicated that the minimum sample size needed to detect such effect is 34 participants (*pwr.t.test* function of the *pwr* package in R). We thus enrolled in the study a convenience sample of 60 participants of which 36 participants were eligible and completed the whole study. This relatively high drop-out rate was due to the need of individual disgust-odor matching (only participants who perceived the study odorants as disgusting could be included to the study), the high requirements of a complex study design (3 study visits stretching over at least 3 weeks) and on participants health status (being free from infection and use of anti-inflammatory drugs for at least fourteen and 10 days, respectively). More information on the selection criteria given below. The resulting group of 36 participants showed a mean age and standard deviation of *M* (STD) = 28 (8.7) years of which 21 female [29.1 (10.8) years] and 15 male [(26.5 (3.9) years] with comparable age (*t*-test, *P* = 0.32) and body mass index BMI [female 21.8 (2), male 22 (2.3), *t*-test, *P* = 0.84]. General health was rated as ‘pretty good’ or ‘very good’ by all participants on a five-point scale ranging from ‘very bad’ to “very good. Mean disgust ratings and distribution of individually chosen disgust odors are given in Table 1.

**Table 1.** List of disgust (lines 1–12) and neutral odorants (lines 13–16), their blending information, and the stimulus disgust they evoked

		Blending fluid	CF	Stimulus disgust (mean)	*N*	Category	Object
1	Isovaleric acid	Mineral oil	0.00016	70.9	7	Body	Smelly feet
2	Pyridine	Mineral oil	0.0001	71.2	1	Body	Gingivitis
3	Skatole	Phtalate	0.01	73.5	10	Body	Feces
4	Sweat, artificial	PG	0.06	65.0	4	Body	Sweat
5	Urine, artificial	PG	0.1	44.2	22	Body	Urine
6	Valeric acid	Phtalate	0.01	65.4	10	Body	Vomit
7	2-Merkaptoethanol	Phtalate	0.001	73.2	25	Food	Rotten egg
8	Butyric acid	Mineral oil	0.00032	71.4	7	Food	Rancid butter
9	Cheese	Pure	/	69.4	19	Food	Old cheese
10	‘Surströmming’	Pure	/	71.6	26	Food	Fermented herring
11	Trimethyl phosphite	Mineral oil	0.00032	70.3	7	Food	Rotten fish
12	Rotten yeast	DW	0.008	65.5	6	Food	Rotten yeast
13	Distilled water (DW)	Pure	/	24.2	36	Neutral	Odor free
14	Mineral oil	Pure	/	20.5	36	Neutral	Odor free
15	Phthalate	Pure	/	21.0	36	Neutral	Odor free
16	Propylene glycol (PG)	Pure	/	22.1	36	Neutral	Odor free

Abbreviations: CF: concentration factor = (volume aliquot/volume mixture).

Participants were recruited via advertisements at Stockholm University, Karolinska Institutet and on student online-platforms. All were offered movie vouchers for their participation. Inclusion criterion were being a non-smoker, reporting being healthy, a functional olfactory sense, and not being pregnant. After giving informed consent, eligible participants were invited for a first screening session to identify for each individual the four most disgusting odors from a sample of twelve. Those four individually selected disgust odors were then to be used during the following exposure session ‘Disgust’ and had to fulfill the requirement that ratings for those four individually most disgusting odors had to be equal to, or exceed, 60 on a disgust visual analogue scales (VAS) ranging from 0 ‘not at all’ to 100 ‘extremely’. Individuals reporting weaker disgust were excluded from the study. This selection procedure was necessary due to the large inter-individual variability in odor evaluation and the need to guarantee successful disgust induction in study participants so as to be able to address our study aim, i.e. to investigate immune system response to olfactory evoked disgust. Participants were informed that the aim of the current study was to investigate how odors, especially unpleasant ones, influence our psychological and physiological well-being. All were given movie vouchers for their participation.

### Stimuli

Sixteen odors were pre-selected in separate pilot studies (not described here): four neutral blending fluids (distilled water, mineral oil, phthalate and propylene glycol) and 12 disgust-odorants comprising complex everyday odorants (fermented herring [a Swedish specialty], French cheese, rotten yeast), monomolecular chemicals (isovaleric acid, pyridine, skatole, valeric acid, 2-mercaptoethanol, butyric acid and trimethyl phosphite), and artificial odorant-mixtures (artificial sweat and artificial urine). In those pilot studies, well-perceivable, iso-intense odor concentrations using appropriate blending fluid were determined. We also decided upon appropriate odor-object identifier that were chosen either from the identity of the real object (fermented herring, French cheese, and rotten yeast), its product name (artificial sweat and urine), descriptions in the literature or from the internet and personal associations of how participants in the pilot studies described the odor qualities (isovaleric acid, pyridine, skatole, valeric acid, 2-mercaptoethanol, butyric acid and trimethyl phosphite; see Table 1). Those odor-object identifiers served as basis for grouping of odorants to the labels ‘body’ or ‘food’. These labels were used immediately before presentation of the odor so as to strengthen the evoked response [22]. Individual ratings of stimulus-induced disgust, given on a VAS from 0 ‘not at all’ to 100 ‘extremely’, obtained after presentation of each odorant, indicated that all disgust odorants were rated as clearly disgusting. However, stimulus-induced disgust varied considerably between odorants, ranging from 44.2 for artificial sweat to 73.5 for skatole (smell of feces, see Table 1).

Column *N* in Table 1 gives the number of participants for whom each odorant was used as one of four individually matched disgust-odorants in the odor exposure session ‘Disgust’. Odorants that were perceived as disgusting by most participants (*N* = 25 and *N* = 26, respectively) were 2-mercaptoethanol (rotten eggs) and fermented herring, whereas only one participant perceived strong disgust from smelling pyridine (gingivitis); indicating considerable individual variation in olfactory perceived disgust between individuals on the one hand, but suggesting conformity of perceived disgust as evoked by rotten food odors.

### Procedure

We used a within-group design requiring two sessions undertaken on separate visits, one for odor exposure ‘Control’ and one for ‘Disgust’ (counterbalanced order). For all visits, participants had to abstain from use of perfume and other fragrances. Odor exposure sessions were at least one week apart (mean 18, min 7, max 66 days) to ascertain immune system normalization after disgust stimulation and were scheduled at standardized times for each participant, either between 9–11.30 a.m. or 12–3 p.m. to avoid confounding with immune system analytes’ diurnal cycles. To warrant good analyte quality, participants could only participate if they reported being free from infection and use of anti-inflammatory drugs for at least 14 and 10 days, respectively, and abstinence from physical exercise for at least 24 h and from alcohol for at least 12 h prior to testing.

Upon arrival for odor exposure sessions, ‘Control’ or ‘Disgust’, a 30-min resting period was executed for familiarization with the new environment and normalization of physiological and immunological parameters. During this time, several psychometric scales were administrated: the Disgust Scale-Revised (DS-R) (here, combining sub-scale measures for ‘Core’, ‘Animal reminder’ and ‘Contagion’ disgust to a total score, with higher scores indicating higher trait disgust [23]); the perceived stress scale measuring trait stress in form of frequency and appraisal of stressful events during the last month with higher scores indicating higher trait stress (PSS10, Cohen & Williamson, 1988); and the perceived vulnerability of disease questionnaire, where higher scores indicated stronger belief in one’s disease susceptibility (PVD) [24]. Questionnaire data were collected as background information and are not reported here. Baseline ratings of self-rated state disgust and state stress were obtained using a paper-pencil VAS, ranging from 0 ‘Not at all’ to 10 ‘Extremely’ disgusted/stressed, and baseline samples of whole saliva were collected.

**Figure 1.**
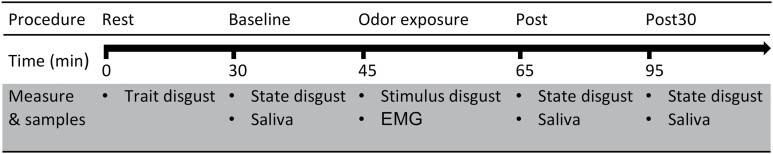
The figure describes an overview of the experimental procedure including the timing of the saliva samples and disgust measurements. EMG = electromyography.

Odor exposure sessions were performed in a quiet well-ventilated room designed for odor perception research purpose, where participants were comfortably seated with his/her chin placed on a chinrest at about 50 cm from a computer screen on which instructions and VAS ratings were presented using E-Prime 2.0 (www.pstnet.com). Odorant presentation was organized in the following way: First, odorant category label (‘Body’ or ‘Food’) were presented, followed by a 5-s countdown for adaptation of breathing pattern and a fixation cross upon which odorants, stored in 160 ml opaque glass jars, were presented by trained investigators quickly opening the jar and placing it in 5 cm distance to the participant’s nose for 3 s of inhalation. After that, participants rated each odorant on 100 step VAS ranging from ‘not at all’ to ‘extremely’ for ‘Intensity’ and ‘Disgust’ ratings, and ranging from ‘extremely unpleasant/-healthy’ to ‘extremely pleasant/healthy’ with ‘neutral’ placed in the middle (50) for ratings of ‘Pleasantness’ and ‘Healthiness’. All odorants were presented in random order with interstimulus intervals of at least 20 s to avoid adaptation and with varying duration due to individual speed of VAS ratings. Each odor exposure session lasted about approximately 20 min with four odorants being presented three times in random order that were unique to each individual (Fig. 1). In the ‘Control’ odor exposure session, the same procedure was followed using four identical odorless blending fluids for all participants, whereas disgusting odorants were individually selected for each participant as a function of individual stimulus disgust ratings from the screening session as described under Participants. After each odor exposure session, measurements (VAS) of state disgust and state stress and saliva sampling were repeated twice, immediately after odor exposure session (post) and 30 min later (post_30_), corresponding to 65 and 95 min after beginning of each odor exposure session. In addition, at time points pre and post_30_, blood samples were taken for analysis of a systemic effect of olfactory evoked disgust on level of IL-6 and blood TNFα in the first 21 participants. Pilot whole blood analyses of this sub-sample (Quantikine HS ELISA) showed no variation with odor exposure. Therefore, blood sampling was discontinued.

### Facial EMG recording

Facial EMG activity was recorded from the corrugator supercilii region located at the forehead above the left eyebrow (frowning), and from the levator labii superiores al nasi region located on the cheek close to the left nose wing (lifting upper lip). Ground electrode was positioned at the forehead border of the hair line, all electrode placement following the recommendation of van Boxtel [25]. After cleaning relevant skin areas with 50% ethanol solution, Al/AgCl surface electrode pairs (recording area 0.4 cm diameter) were filled with electrode gel (Signa gel, Parker, www.cortechsolutions.com) and mounted to target areas. EMG signal was recorded using AD Instruments differential BioAmp amplifier controlled by a PC using LabChart7 pro software (www.ADInstruments.com) with the following settings: input impedance of 200 MΩ, amplification range +/– 5 µV to +/100 mV, gain accuracy +/1.5%, common mode rejection ratio 85 dB @ 60 Hz, 1000 Hz sampling rate, mains filter, first-order 10 Hz high-pass filter, and fourth-order Bessel 500 Hz low-pass filter at –3 dB [26]. Offline data processing for statistical analysis comprised 20 Hz high-pass filtering, down sampling to 100 Hz, rectifying and smoothing with a window size of 300 ms [27]. In a next step, mean values were calculated separate for stimulus category (disgust, neutral, food and body) over the following time windows: 1 s baseline (at 5–4 s before olfactory stimulation), 12 target time-windows (500 ms, ranging from 3 s before to 3 s after olfactory stimulation). For baseline correction, target/baseline ratios were calculated [25] and outlier were removed separately for disgust and neutral trials by replacing with the stimulus category mean in case of deviation exceeding three standard deviations from the mean.

### Saliva sampling and measurement of TNFα and sIgA

Two salivary immune markers were measured in the current study: Tumor necrosis factor α(TNFα) and secretory immunoglobulin A (sIgA). To do so, 3 mL whole saliva samples were collected using passive drool technique through clean 5 cm plastic straws into 10 ml screw-lid plastic centrifuge test tube vials while recording sampling time for later correction for salivary flow. Participants were instructed to imagine eating their favorite dish to increase salivary flow, and to then carefully extrude the accumulating saliva with their tongue into the straw. After sampling, saliva was centrifuged, aliquoted, bar-code labeled, and frozen to –80 °C within one hour. After collecting all samples from all participants, saliva aliquot was thawed for analysis of TNFα and sIgA using Invitrogen’s UltraSensitive TNFα (KHC3013) and Salimetrics’ salivary secretory IgA (1-1602) enzyme immunoassay kits, respectively, and results were reported in pg/mL for TNFα and in ug/mL for sIgA. Analyses with ELISA were performed in duplicate; thus 432 data points were available per measure [36 participants * 2 odor exposure sessions (control vs. disgust) * 3 time points (baseline, post, post_30_) * 2 ELISA analyses].

Levels of TNFα and sIgA corresponded well with reports from earlier studies [28] and lay well above the detection limit of the Elisa kits: for TNFα 0.09 pg/mL, for sIgA 2.5 µg/mL (see Invitrogen’s salivary TNFα ELISA kit KHC3013 and Salimetrics’ sIgA ELISA kit 1-1602 for more information). All data were log-transformed before analysis to better approximate a normal distribution and secretory flow rates (pg/min for TNFα and ug/min for sIgA) were calculated to correct for the impact of salivary flow (saliva volume/ sampling time), as recommended [29].

### Analysis

The following analyses were performed using IBM-SPSS 22: Baseline-corrected scores of state disgust and stress were used for analysis and repeated measures ANOVAs were calculated including the within-subject factors ‘Odor condition’ (disgusting vs neutral odorants) and ‘Time’ (pre, post and post_30_).

Physiological (EMG) data were analyzed using mixed model analyses. A mixed effect approach was used including the fixed factor ‘Odor condition’ and ‘Time’ and the random factor ‘Subject’, allowing intercepts to vary between participants. A stepwise elimination procedure was followed by a chi-square test comparing a model with and without the critical effect and those effects significantly improving the model fit were kept and effect size b reported.

To examine whether the levels of immunological markers (TNFα, sIgA) were influenced by the odor condition we used a Linear Mixed-Effects Model, LMM (*lmer* function in the *lme4* R package). Our dependent variable was either TNFα or sIgA levels. Our exploratory variables were ‘Odor condition’ (neutral vs. disgusting odor), ‘Time’ (baseline, post, and post_30_) and their interaction. Finally, we included a random intercept for each subject’s ID and random slopes for odor condition by subject (time by subject was initially added but the models did not converge and had to be removed). We also controlled for the robustness of our results by doing the same models but controlling for several confounding factors such as sex, BMI, and disgust sensitivity (DSR total score).

Based on reviewers’ comments we also completed a Linear Mixed-Effects Model exploring how the levels of immunological markers (dependent variables) are influenced by ‘Time’ (baseline, post, and post_30_) within each condition. In the latter model, we included a random intercept for each subject’s ID and random slopes for time by subject.

Mixed models were performed using R, version 4.1.3.

## RESULTS

### State measures of disgust and stress

**Figure 2.**
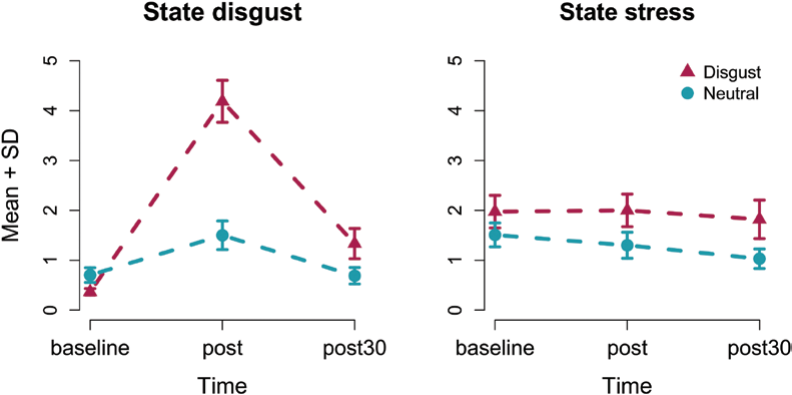
Ratings of (a) state disgust and (b) stress are given on a 0 to 10 visual analogue scale (VAS) at three time points (minutes): before (pre) directly after (post) and 30 min after exposure to disgusting or control odors (post30). We find a significant interaction in form of stronger relative increase of state disgust in disgust than control condition from time point pre to post. No such significant interaction effect was found for state stress. SEM: standard error of mean.

Ratings of state disgust (Fig. 2) showed a significant interaction in form of stronger increase at time point Post following Disgust as compared to Control exposure (*F*_(1,35)_ = 27, *P* < 0.01), in addition to significant main effects of Time and Odor condition (*F*_(1,35)_ = 56, *P* < 0.01 and *F*_(1,35)_ = 40, *P* < 0.01, respectively). These results confirm our intended manipulation of olfactory evoked disgust which was most evident at time point Post but that had dissipated by time point Post_30_.

Levels of state stress, however, differed only marginally between Control and Disgust conditions with corresponding means (and SD) at time point Pre being 1.5 (1.4) on a 10-point scale versus 2.0 (2.0), at time point Post being 1.3 (1.6) versus 2.0 (2.0), and at time point Post_30_ being 1.0 (1.2) versus 1.8 (2.3). Repeated measures ANOVA on baseline-corrected state stress confirmed this impression showing no significant changes, over time, between exposures, and no significant interaction either (all Fs_(1,35)_ < 2 and *p*s >.10).

### EMG measures

**Figure 3.**
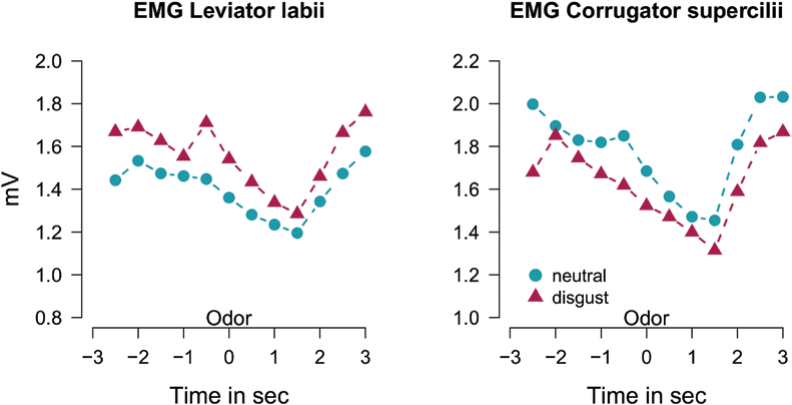
Electromyography (EMG) response recorded at the levator labii (3a) and corrugator supercilii region (3b) are given for the time 6 s around odorant presentation (0 s). Baseline corrected mean levels for 12 time windows (each 0.5 s duration) are given from ‘Disgust’ and ‘Neutral’ exposure conditions showing larger EMG amplitudes during Disgust exposure for the levator labii region and during Neutral exposure for the corrugator supercilii region.

At the levator labii region, mean EMG amplitudes over the whole –3 to 3 s time window were significantly increased during exposure to disgusting odors (*χ*^2^_(1)_ = 252.2, *P* < 0.001) with effect size *b* = 0.16 and standard error SE = 0.07 (Fig. 3). This overall higher muscular activity both pre- and postexposure, probably relates to a generalized state of disgust, evoked during the 20 minutes disgust exposure session. This assumption is supported by increased ratings of state disgust as reported in Section 3.1 and Fig. 2. EMG amplitudes increased in response to disgust odors already at time window –0.5 to 0 s (Fig. 3). At this time point, the odor containing glass jar had already been opened and was being placed 5 cm from the participant’s nose ready for inhalation at time point 0. This pre-activation could thus indicate that the odor stimuli reached participants olfactory receptors already at this time window (–0.5 to 0 s) or it could indicate a preparatory response in expectation of the next odor. Analysis of mean EMG amplitude in this particular time window approached significance (χ^2^_(1)_ = 3.6, *P* < 0.057, *b* = –0.076, SE = 0.03) indicating enhanced increase of levator labii activation during disgust odor inhalation as compared to neutral ones.

Investigation of the corrugator supercilii region revealed a significant effect of exposure but this time larger EMG amplitudes occurred after exposure to neutral odors (χ^2^_(1)_ = 90.4, *P* < 0.001, effect size *b* = –0.15, SE = 0.09). Possibly, this increased activation with neutral odors may be related to higher sniff vigor during ‘Control’ conditions due to the neutral odorants’ relatively weaker or less unpleasant percept [30]. The same follow-up mixed model calculated for levator labii activation at time window –0.5 to 0 s (inhalation) showed no significance at time point of odor presentation (χ^2^_(1)_ = –0.146, *P* < 0.70, *b* = –0.019, SE = 0.05).

### TNFα and sIgA as a function of disgust exposure

**Figure 4.**
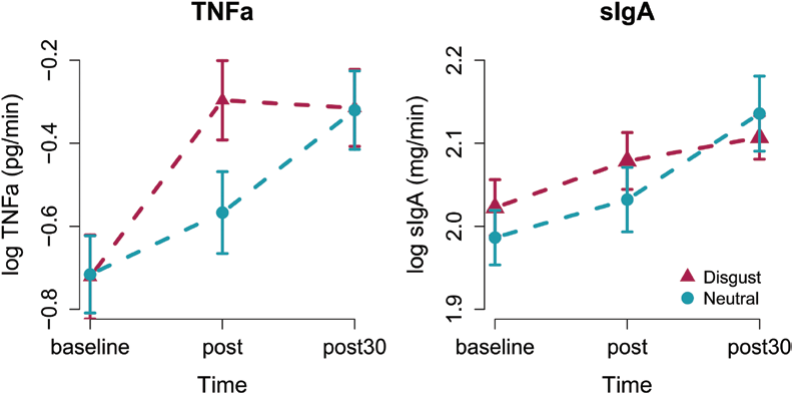
(a) Salivary TNFα and (b) sIgA levels, sampled before (baseline) directly after (post) and 30 min after exposure to disgusting or control odors (post30). Mean values and standard error of means are given for logarithmic data corrected for salivary secretion rate. We find a significant interaction for TNFα in form of stronger relative increase of TNFα levels in disgust than control condition from time point pre to post.

The LMM for TNFα showed significant main effects of Time from pre to post (*b* = 0.15, SE = 0.07, *t*_(1,360)_ = 2.17, *P* = 0.03) and from pre to post_30_ (*b* = 0.40, SE = 0.07, *t*_(1, 360)_ = 5.77, *P* < 0.001; see Fig. 4 and Fig. S1 for individual datapoints across time and per condition) but no main effect of Odor condition was found (*b* = –0.01, SE = 0.13, *t*_(1, 56)_ = –0.05, *P* = 0.96). Moreover, we found a significant interaction between Time and Odor condition from pre to post (*b* = 0.28, SE = 0.10, *t*_(1, 360)_ = 2.86, *P* = 0.005) showing a stronger rise in TNFα levels after disgust as compared to neutral odor exposure at post but not at post_30_ (*b* = 0.01, SE = 0.1, *t*_(1, 360)_ = 0.12, *P* = 0.90). Corresponding analysis of sIgA showed significant increase of sIgA only at time point post_30_ (*b* = 0.15, SE = 0.02, *t*_(1, 360)_ = 6.30, *P* < 0.001) but with no effect of Odor condition or interaction between Time*Odor condition (all *P*-values > 0.05). The results found were robust to the inclusion of the confounding variables (sex, BMI, DSR) and none of the confounding variables significantly influenced the outcome variables (all *P*-values > 0.30).

The LMM exploring how Time affects levels of the immunological markers within each condition revealed that TNFα levels significantly increased from pre to post (neutral: *b* = 0.15, SE = 0.06, *t*(1,36) = 2.55, *P* = 0.02; disgust: *b* = 0.43, SE = 0.09, *t*(1,36) = 4.96, *P* < 0.0001) and pre to post30 (neutral: *b* = 0.40, SE = 0.13, *t*(1,206) = 3.091, *P* = 0.004; disgust: *b* = 0.41, SE = 0.10, *t*(1,36) = 4.21, *P* < 0.001). IgA levels significantly increased within both conditions from pre to post30 (neutral: *b* = 0.15, SE = 0.05, *t*(1,36) = 3.005, *P* = 0.005; disgust: *b* = 0.08, SE = 0.03, *t*(1,206) = 2.91, *P* = 0.006) but not from pre to post (neutral: *b* = 0.05, SE = 0.03, *t*(1,36) = 1.32, *P* = 0.20; disgust: *b* = 0.06, SE = 0.03, *t*(1,36) = 1.78, *P* = 0.08).

## DISCUSSION

### Effects of olfactory evoked disgust on oral immunity

The purpose of this study was to investigate whether olfactory evoked disgust is able to activate an oral immune response, thereby confirming interaction between mechanisms of pathogen avoidance (disgust) and pathogen elimination via the immune system.

Our finding that salivary TNFα levels increase upon olfactory evoked disgust corresponds well with the consistent findings reported [4, 5, 31] showing increased salivary TNFα levels after visually evoked disgust. Because TNFα has a role in both pathogen defense and chemosensory perception [19, 32], the increase of salivary TNFα following exposure of disgusting odors can be interpreted as fulfilling two purposes: First, mounting an oral immune response for pathogen disposal, and second, increasing bitter taste sensitivity to avoid further pathogen ingestion [19, 32]. Thereby, the TNFα response can be a mechanism linking the distaste/disgust-mediated pathogen avoidance and the immune system-mediated pathogen elimination mechanisms. It might also have a potential broader impact on nutrition control. Interestingly, TNFα expressing taste buds themselves are sensitive for perception of sweet and umami whereas bitter taste receptors were reported to express another cytokine, the immune suppressive interleukin 10 (IL-10) and a regulating mechanism has recently been described between these receptor types [18].

Behavioral conditioning of immune functions can potentially explain how oral immune markers are produced upon smelling of disgusting odors [33]: A substance without effect on the immune system (conditioned stimulus, CS) can acquire immunomodulating properties when administered together with an immunomodulation agent (unconditioned stimulus, US). Often the CS that will acquire immunomodulatory properties has been a taste, odor, or flavor, i.e. a combination of taste and odor [34]. Indeed, animal studies have shown the reliability of this chemosensory-immune learning phenomenon [35] as well as identifying neurobiological mechanisms mediating this effect, especially involving the insula and amygdala regions [36]. Associative learning might explain how a broad variety of odors without direct pathogen relationships can acquire the potency to modulate oral immunity, simply by being presented together with an immunomodulatory agent.

The effect of disgust stimuli on sIgA seems more complex than previously thought. Whereas [4, 5] found negative effects of visual disgust on sIgA, we found no significant effect of olfactory evoked disgust on sIgA levels. In addition, a recent study shows an increase of sIgA after exposure to disease-related disgust videos [37]. Such discrepancy can, of course, have several reasons. One possibility is that sIgA and TNFα vary in their sensitivity to other confounding factors, such as disgust trait sensitivity [4, 5, 31], general mood [38] and parallel stimulation of taste [39].

Moreover, visually and olfactory evoked disgust may have differing effects on the salivary immune response because odors, but not pictures, directly activate the salivary reflex [40] which results both in differing activation pattern of the parasympathetic and sympathetic branch of the ANS [22] and in divergent activation of the different salivary gland types that may result in different salivary compositions [40, 41].

Although our sample size is higher than several previous published studies that found a significant increase of immuno-markers after exposure of disease-related stimuli, our achieved sample size is relatively modest. Based on [5], a power analysis indicated that our sample size would be large enough to detect a similar effect. It needs also to be mentioned that the effect size observed in our data when performing a paired *t*-test comparing the difference pre to post of TNFα levels between the two conditions (neutral vs disgust) is *d* = 0.52 (*cohen’s d* function of the *effect size* package in R), thus higher than the minimal theoretical effect size (*d* = 0.48) we would be able to detect for a similar test with our achieved sample size (*n* = 36) at 80% power.

As noted, for a subset of participants (*n* = 21) blood samples were taken for analysis of a systemic effect of olfactory evoked disgust on level of IL-6 and blood TNFα that showed no variation with odor exposure. Future studies should investigate whether disgust stimuli drive systemic and local immune responses differently depending on the sense modality used for triggering the experience of disgust.

### Olfactory evoked disgust

We found large inter-individual variability in stimulus disgust (mean disgust ratings of individual odors between 44.2 and 73.5 on 100 point VAS) and state disgust ratings (disgust induction in odor exposure session disgust *M* = 3.8, STD = 2.5 on 10 point VAS), which made it necessary to use individually matched disgust odors. We believe this led to a distinct manipulation of olfactory disgust between odor conditions. Difficulties in evoking olfactory disgust have been reported by others, resulting in the need to provide additional cues together with disgust odors in order to enhance ratings of olfactory evoked disgust [22]. These findings are in line with other work on odor perception, notably the high inter-individual variability in hedonic response and the general susceptibility to contextual cues (e.g., name labels), which modulate odor pleasantness [42, 43]. With this background, it would be interesting for future studies to look more closely at perceptually ambiguous odorants to see whether the same odorant could trigger different immune responses just by changing its label or if the immune response is more associated to the odorant itself.

We used EMG as an objective measure of olfactory evoked disgust and found an overall increased activation in the levator labii region during disgust as compared to control odor exposure. This finding, in parallel to subjective disgust ratings, further confirmed that our experimental design successfully manipulated odor-evoked disgust. This observation is well in line with other studies that often choose the levator labii region as the only EMG indicator of distaste and disgust [44]. In our study, EMG activity of the corrugator supercilii region was decreased during disgust as compared to control exposure, calling into question measures of corrugator supercilii as a reliable indicator of disgust. These findings correspond to a study suggesting comparable decrease of activation in forehead musculature (medial frontalis) upon exposure to body odors of from disgusted individuals [45]. Instead of indicating disgust, supercilii activation may vary with sniff vigor which has been shown to differ in relation to odor pleasantness [30]. Moreover, earlier studies failed to show susceptibility for induced disgust in corrugator supercilii muscle region [13, 14] and typically report the corrugator supercilii region as mainly responsive to overall odor unpleasantness [10, 46].

It is important to note that disgust odor exposure did not significantly alter the level of perceived stress in our volunteers. Stress, being a well-established behavioral effector on the immune system [47], is therefore not a likely candidate to have contributed to changes of TNFα levels in the present study.

## CONCLUSION

We show that individually tailored odors can be potent stimuli to evoke state disgust in healthy volunteers. Interestingly, disgusting smells induced increased levels of the pro-inflammatory cytokine TNFα. These results are a first demonstration of chemosensory pathogen avoidance, in the form of disgust, being linked to immunological pathogen elimination. Altogether the results indicate that human olfaction, akin to animal studies, may have an active part in the defense against disease; not only as a keen detector of early sickness cues but also in launching a preparatory immune response in response to disgusting stimuli.

## Supplementary Material

eoac042_suppl_Supplementary_Figure_S1
