# Supplementary material for: Disgusting odors trigger the oral immune system
Source: Evol Med Public Health. 2022 Dec 15;11(1):8–17. doi: 10.1093/emph/eoac042 (PMC9912705; doi:10.1093/emph/eoac042)
Supplement: eoac042_suppl_Supplementary_Figure_S1 [file eoac042_suppl_supplementary_figure_s1.docx]

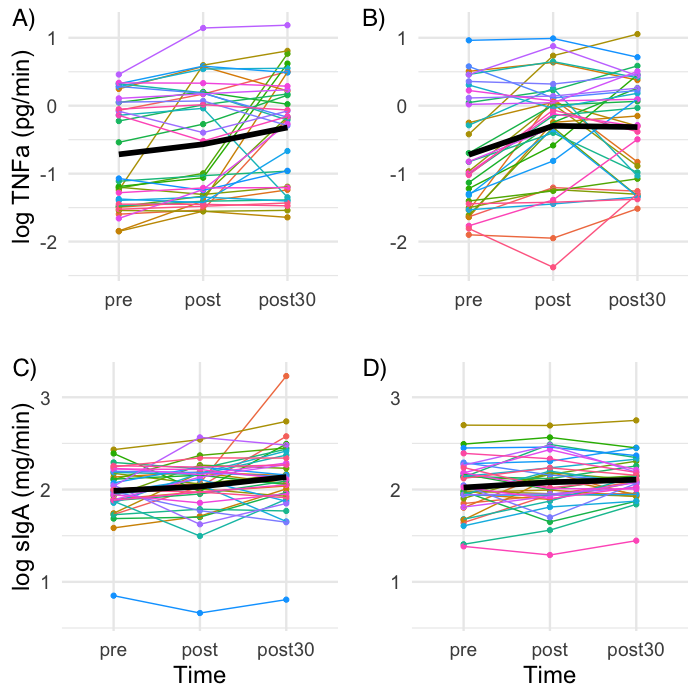


**Figure S1.** Variations of TNFα (A & B) and sIgA (C & D) levels across time (baseline, post, post30) for neutral (left) and disgust (right) conditions. Each colored line represents a single participant. The thick black line represents the average levels across participants (also seen in Figure 4).
